# Level of obesity is directly associated with the clinical and functional consequences of knee osteoarthritis

**DOI:** 10.1038/s41598-020-60587-1

**Published:** 2020-02-27

**Authors:** Benjamin Raud, Chloé Gay, Candy Guiguet-Auclair, Armand Bonnin, Laurent Gerbaud, Bruno Pereira, Martine Duclos, Yves Boirie, Emmanuel Coudeyre

**Affiliations:** 1 Service de Médecine Physique et de Réadaptation, CHU Clermont-Ferrand, unité de nutrition humaine, INRA, Université Clermont Auvergne, F-63003 Clermont-Ferrand, France; 2 Service de santé publique, CHU Clermont-Ferrand, F-63003 Clermont-Ferrand, France; 3 Université Clermont Auvergne, CNRS, SIGMA Clermont Institut Pascal, F-63000 Clermont Ferrand, France; 4 Délégation Recherche Clinique et Innovation, CHU Clermont-Ferrand, 63003 Clermont-Ferrand, France; 5 Service de Médecine du Sport et explorations fonctionnelles, CHU Clermont-Ferrand, unité de nutrition humaine, INRA, Université Clermont-Auvergne, F-63003 Clermont-Ferrand, France; 6 Service de Nutrition Clinique, CHU Clermont-Ferrand, unité de nutrition humaine, INRA, Université Clermont Auvergne, 63003 Clermont-Ferrand, France

**Keywords:** Epidemiology, Cartilage

## Abstract

Obesity is one of the most important risk factors of knee osteoarthritis (KOA), but its impact on clinical and functional consequences is less clear. The main objective of this cross-sectional study was to describe the relation between body mass index (BMI) and clinical expression of KOA. Participants with BMI ≥ 25 kg/m^2^ and KOA completed anonymous self-administered questionnaires. They were classified according to BMI in three groups: overweight (BMI 25–30 kg/m^2^), stage I obesity (BMI 30–35 kg/m^2^) and stage II/III obesity (BMI ≥ 35 kg/m^2^). The groups were compared in terms of pain, physical disability, level of physical activity (PA) and fears and beliefs concerning KOA. Among the 391 individuals included, 57.0% were overweight, 28.4% had stage I obesity and 14.6% had stage II/III obesity. Mean pain score on a 10-point visual analog scale was 4.3 (SD 2.4), 5.0 (SD 2.6) and 5.2 (SD 2.3) with overweight, stage I and stage II/III obesity, respectively (p = 0.0367). The mean WOMAC function score (out of 100) was 36.2 (SD 20.1), 39.5 (SD 21.4) and 45.6 (SD 18.4), respectively (p = 0.0409). The Knee Osteoarthritis Fears and Beliefs Questionnaire total score (KOFBEQ), daily activity score and physician score significantly differed among BMI groups (p = 0.0204, p = 0.0389 and p = 0.0413, respectively), and the PA level significantly differed (p = 0.0219). We found a dose–response relation between BMI and the clinical consequences of KOA. Strategies to treat KOA should differ by obesity severity. High PA level was associated with low BMI and contributes to preventing the clinical consequences of KOA.

## Introduction

Osteoarthritis (OA) is the most common joint disease and one of the most prevalent symptomatic health problems^1^. Knee osteoarthritis (KOA) leads to knee pain and altered joint function, with socioeconomic consequences^2^. It generates a high proportion of health costs in many countries and has become a major public health issue. The health costs are directly related to KOA, such as knee replacement, or substantially by medication consumption^3^.

In the many studies investigating the risk factors of KOA, overweight and obesity remain the most determinant even though they are considered modifiable. A recent meta-analysis^4^ showed a 5-unit increase in body mass index (BMI) associated with a 35% increased risk of KOA (relative risk [RR]: 1.35; 95% confidence interval [CI]: 1.21–1.51). BMI was positively associated with increased risk of KOA defined by plain radiography and/or clinical symptoms (RR: 1.25, 95% CI: 1.17–1.35) and clinical surgery (RR: 1.54, 95% CI: 1.29–1.83). Another study suggested a longitudinal association between weight gain and increased risk of symptomatic OA^5^. The Framingham study showed an association of decreased BMI by ≥2 units at 10 years before examination and 50% decreased risk of OA for women^6^. In another cohort study^7^, a weight loss of >10% could reduce the clinical consequences of OA, finding a dose–response association between weight loss and pain or articular function.

Overweight and obesity are well known to increase the risk of KOA by mechanical load on weight-bearing joints^8^. However, obesity or metabolic syndrome also increase the risk of hand OA^9^. Hence, metabolic diseases, such as diabetes or metabolic syndrome, could have systemic effects on joints. A recent meta-analysis reported that type 2 diabetes mellitus may be a risk factor for OA whatever the location^10^. Few studies have explored the association between obesity stage and KOA consequences on disability. A recent study showed that waist circumference could be one of the main risk factors for limiting ambulation speed in adults with KOA^11^.

Regular physical activity (PA) as well as caloric restriction can reduce the clinical consequences of KOA^12^ and potentially contribute to weight loss. However, the impact of BMI on level of PA in people with OA is unknown. A recent study measured the level of PA in a normal-weight population versus unhealthy and healthy overweight and obese participants. PA was lower for unhealthy overweight and healthy and unhealthy obese participants than healthy overweight and normal-weight participants^13^. More specifically, a recent meta-analysis found that people with KOA were the least active according to PA guidelines^14^. Other studies of KOA suggest that being overweight or obese is associated with lower quality of life and higher risk of disability^15^ and may affect knee joint impact rates and cause incremental pain^16^. Also, overweight and obesity are risk factors for pain in the global population^17^.

Despite obesity being a risk factor for KOA, we have few data on the association of obesity severity and its clinical and functional consequences. This study aimed to describe the association between KOA and BMI gradation in terms of pain, physical disability, level of PA and fears and beliefs concerning KOA.

## Methods

This study is part of a larger cross-sectional study of people with KOA older than 18 years of age that took place in France between September and November 2014 in 9 spa therapy resorts dedicated to OA. Every thermal establishment provided identical care for the patients, and procedures were similar for each center. For each patient, OA was the indication that led to prescribing spa therapy.

The study protocol was approved by the ethics committee of the university hospital of Clermont-Ferrand (medical ethics committee of South-East France Sud-Est 6, authorization no.: 90 2015/CE38) and was registered at ClinicalTrials.gov (NCT02681133). It was conducted in compliance with the Good Clinical Practices protocol and Declaration of Helsinki principles. All participants gave their verbal consent to participate after being informed about the study protocol.

Individuals were recruited on a voluntary basis and were included if they had symptomatic KOA (diagnosis of KOA confirmed by a physician, according to the criteria of the American College of Rheumatology^18^). Individuals younger than 18 years of age, with behavioral and comprehension difficulties and with bilateral total knee replacement were excluded. For the purpose of this study, data for only people with BMI ≥ 25 kg/m^2^ were retained for analysis.

Participants were classified according to their BMI in three groups: overweight (BMI 25–30 kg/m^2^), stage I obesity (BMI 30–35 kg/m^2^) and stage II/III obesity (BMI ≥ 35 kg/m^2^).

### Data collection

Height and weight to calculate the BMI of each participant were measured by the physician in charge of the patient at inclusion. Other data were collected by use of an anonymous self-administered questionnaire. Posters were placed in each spa therapy resort to inform participants that research staff were available to help them complete the questionnaire if needed. Participants could complete their questionnaire at any time during their stay in the resort.

We collected sociodemographic data (sex, age) and clinical data: OA duration, joint replacement (knee and/or hip), and comorbidities (diabetes, hypertension, renal failure, gastrointestinal bleeding, anxiety/depression, physical impairment limiting activity, cardiovascular disease) by declarative information based on Osteoarthritis Research Society International guidelines^12^. To avoid any misdeclaration, the pharmacological treatment reported by the participant was considered.

Pain during the last day and the most intense pain during the last month were assessed by a visual analog scale [VAS], from 0, no pain, to 10, very severe pain. Participants reported whether they were receiving treatment for OA pain and if they had another painful joint.

PA level was assessed by the short form of the International Physical Activity Questionnaire (IPAQ)^19^. We estimated continuous scores in metabolic equivalent minutes per week (MET-min/week) for vigorous, moderate, walking and total activity, as PA level (low, moderate or high).

The Western Ontario and McMaster Universities Osteoarthritis Index (WOMAC) was used to assess function in terms of physical disability. Only the function sub-scale was evaluated. The scale consists of 17 items that are summed to give a score, which was normalized to a 0 to 100 score. Higher scores indicate more severe impairment^20^.

Fears and beliefs about KOA were assessed by the 11-item Knee Osteoarthritis Fears and Beliefs Questionnaire (KOFBeQ). Five scores were estimated: a total score and 4 sub-scores for fears and beliefs about activities of daily living, physicians, disease, and sports and leisure activities. Higher scores indicate greater fears and beliefs^21^.

### Statistical analysis

Statistical analysis involved using SAS v9.4. Two-tailed P <0.05 was considered statistically significant. No imputation method was used to replace missing data. Continuous data are presented as mean (SD) and categorical data as number (%). The three BMI groups (overweight, stage I obesity, stage II/III obesity) were compared in terms of sex, age, BMI, OA duration, joint replacement and comorbidities by the non-parametric Kruskal-Wallis test for continuous factors and chi-square or Fisher exact test for categorical factors. Generalized linear mixed models were used to compare the three BMI groups in terms of pain, WOMAC function score, IPAQ scores and KOFBeQ scores, with spa therapy resorts as a random effect and adjusted on potential confounders sex, age and number of comorbidities.

## Results

### Description of participants

**Figure 1:**
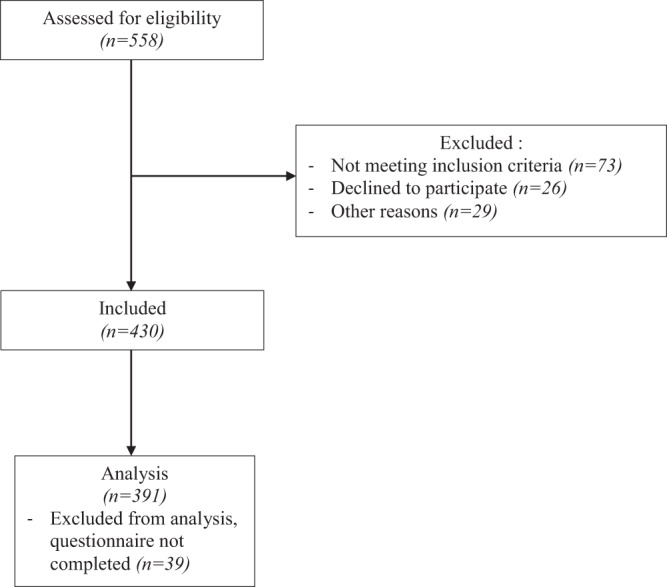
Flow diagram of participants in the study.

We included 391 individuals with BMI ≥ 25 kg/m^2^ (Fig. 1): 223 (57.0%) were overweight, 111 (28.4%) had stage I obesity, and 57 (14.6%) had stage II/III obesity. The characteristics of participants are described in Table 1. BMI groups did not differ by sex, age, OA duration and joint replacement.

**Table 1:** Characteristics at baseline for individuals with knee osteoarthritis (OA).

	Overweight	Stage 1	Stage II/III	Total	P value
	n = 223	n = 111	n = 57	n = 391	
**Sex**, n (%)					*0.1188*
Male	70 (31.4)	32 (28.8)	10 (17.5)	112 (28.6)	
Female	153 (68.6)	79 (71.2)	47 (82.5)	279 (71.4)	
**Age** (years), mean (SD)	67.8 (8.1)	67.4 (7.5)	65.4 (7.5)	67.3 (7.9)	*0.1566*
**BMI** (kg/m^2^), mean (SD)	27.2 (1.3)	32.0 (1.3)	39.6 (5.9)	30.4 (5.0)	*<0.0001*
**OA duration** (years), mean (SD)	12.4 (10.4)	12.4 (9.8)	14.1 (10.6)	12.7 (10.2)	*0.3320*
**Joint replacement (knee and/or hip)**, n (%)	40 (18.5)	26 (24.5)	10 (19.6)	76 (20.4)	*0.4483*
Total knee replacement	22 (10.2)	15 (14.2)	6 (11.8)	43 (11.5)	
Total hip replacement	18 (8.3)	11 (10.4)	4 (7.8)	33 (8.8)	
**Traumatic or surgery knee history**, n (%)	91 (42.3)	45 (43.7)	26 (47.3)	162 (43.4)	*0.8025*
**Comorbidities**, n (%)					
Diabetes	21 (9.4)	23 (20.7)	11 (19.3)	55 (14.1)	*0.0094*
Hypertension	77 (34.5)	57 (51.4)	32 (56.1)	166 (42.5)	*0.0011*
Renal failure	7 (3.1)	0	1 (1.8)	8 (2.0)	*0.1608*
Gastrointestinal bleeding	10 (4.5)	11 (9.9)	6 (10.5)	27 (6.9)	*0.0928*
Anxiety/depression	32 (14.3)	20 (18.0)	17 (29.8)	69 (17.6)	*0.0236*
Physical impairment limiting activity	29 (13.0)	15 (13.5)	9 (15.8)	53 (13.6)	*0.8604*
Cardiovascular disease	41 (18.4)	20 (18.0)	8 (14.0)	69 (17.6)	*0.7386*
**Number of comorbidities**, mean (SD)	1.1 (1.1)	2.4 (1.2)	2.6 (1.1)	1.7 (1.3)	*<0.0001*

The mean number of comorbidities was 1.1 (SD 1.1) for overweight people as compared with 2.4 (SD 1.2) and 2.6 (SD 1.1) for stage I and stage II/III obesity groups (p<0.0001). The BMI groups significantly differed in terms of comorbidities for diabetes, hypertension and anxiety or depression: 9.4%, 20.7% and 19.3% of the overweight, stage I and stage II/III groups, respectively, were followed up for diabetes (p = 0.0094); 34.5%, 51.4% and 56.1%, respectively, reported hypertension (p = 0.0011); and 14.3%, 18% and 29.8%, respectively, reported anxiety or depression (p = 0.0236). The BMI groups did not differ in terms of renal failure, gastrointestinal bleeding, physical impairment limiting activity, or cardiovascular disease.

According to the Osteoarthritis Research Society International (OARSI) phenotypes^12^, 96.5% of stage II/III participants had polyarthritis with comorbidities profiles, as compared with 90.1% of stage I and 57.8% of overweight participants (p < 0.0001).

### Pain

After adjustment for sex, age and number of comorbidities, pain intensity during the last 24 hours increased significantly with BMI gradation (p = 0.0367) (Table 2). For the most intense pain during the last month, only overweight and stage II/III groups differed significantly. Pain intensity during the last 24 hours was >4/10 for 46.8% of overweight individuals versus 60% for stage I individuals (p = 0.0493) and 65.5% for stage II/III individuals (p = 0.0343). Overall, 79.6% of participants with stage II/III obesity reported receiving treatment for pain due to OA versus 68.3% and 65.6% of stage I and overweight participants (p = 0.4199).

**Table 2:** Description of pain reported by participants.

	Overweight	Stage I	Stage II/III	Total	P value^*^		
					Stage I vs Overweight	Stage II/III vs Overweight	Stage II/III vs Stage I
**Pain during the last 24 hours** (VAS^†^ 0–10), mean (SD)	4.3 (2.4)	5.0 (2.6)	5.2 (2.3)	4.7 (2.5)	*0*.*0356*	*0*.*0462*	*0*.*7454*
**Most intense pain during the last month** (VAS 0–10), mean (SD)	6.4 (2.3)	6.8 (2.4)	7.4 (1.9)	6.7 (2.3)	*0*.*2973*	*0*.*0329*	*0*.*2241*
**Treatment for pain due to OA**, n (%)	139 (65.6%)	71 (68.3%)	43 (79.6%)	253 (68.4%)	*0*.*9807*	*0*.*2034*	*0*.*2286*
**Other painful joint**, n (%)							
Lumbar spine	154 (69.1%)	80 (72.1%)	38 (66.7%)	272 (69.6%)	*0*.*8967*	*0*.*4596*	*0*.*4386*
Cervical spine	129 (57.8%)	57 (51.4%)	36 (63.2%)	222 (56.8%)	*0*.*1518*	*0*.*4619*	*0*.*0939*
Hands	109 (48.9%)	44 (39.6%)	29 (50.9%)	182 (46.5%)	*0*.*0673*	*0*.*8041*	*0*.*2804*
Shoulders	107 (48%)	60 (54.1%)	29 (50.9%)	196 (50.1%)	*0*.*4979*	*0*.*9994*	*0*.*6333*
Hips	68 (40.5%)	43 (38.7%)	23 (40.4%)	134 (34.3%)	*0*.*1924*	*0*.*2628*	*0*.*9281*

^*^Generalized linear mixed models with spa therapy resorts as a random effect and adjusted for sex, age and number of comorbidities.

^†^VAS: visual analog scale (0, no pain; 10, very severe pain).

Participants reported another painful joint (Table 2), with no significant difference between groups. Multiple-joint OA was reported by 91.5%, 90.1% and 96.5% of participants in overweight, stage I and stage II/III obesity groups, respectively.

### Physical disability

The WOMAC function scores are described in Table 3. The BMI groups significantly differed in physical disability (p = 0.0409). The stage II/III obesity group was significantly more impaired than the overweight group (p = 0.0115).

**Table 3:** Description of WOMAC, IPAQ and KOFBeQ scores.

	Overweight	Stage 1	Stage II/III	Total	P value^*^		
					Stage I vs Overweight	Stage II/III vs Overweight	Stage II/III vs Stage I
**WOMAC function score** (0–100), mean (SD)	36.2 (20.1)	39.5 (21.4)	45.6 (18.4)	38.5 (20.4)	*0.4468*	*0.0115*	*0.0703*
**IPAQ physical activity level**, n (%)					*0.3770*	*0.0058*	*0.0523*
Low	40 (18.5)	18 (17.3)	15 (27.3)	73 (19.5)			
Moderate	75 (34.7)	46 (44.2)	27 (49.1)	148 (39.5)			
High	101 (46.8)	40 (38.5)	13 (23.6)	154 (41.1)			
**IPAQ score** (MET-min/week), mean (SD)							
Vigorous activity^†^	2 956.8 (2 388.7)	2 472.0 (1 945.8)	2 653.3 (2 679.6)	2 803.7 (2 297.0)	/	/	/
Moderate activity	1703.2 (1338.7)	1829.1 (1543.6)	1100.0 (1010.1)	1645.0 (1368.6)	*0.8521*	*0.0020*	*0.0035*
Walking	1373.4 (1116.5)	1357.9 (1261.1)	942.1 (1001.5)	1312.1 (1149.6)	*0.4067*	*0.0053*	*0.0403*
Total activity	3627.5 (2926.4)	3249.7 (2602.3)	2094.6 (1832.1)	3292.7 (2745.2)	*0.1617*	*<0.0001*	*0.0040*
**IPAQ time spent sitting** (min/week), mean (SD)	276.5 (141.7)	283.5 (132.7)	351.4 (186.2)	289.8 (149.0)	*0.4970*	*0.0006*	*0.0072*
**KOFBeQ scores**, mean (SD)							
Total score	41.5 (22.7)	45.9 (23.1)	52.4 (20.6)	44.4 (22.8)	*0.1843*	*0.0064*	*0.1248*
Activities of daily living	8.8 (7.7)	9.9 (8.4)	12.4 (8.5)	9.6 (8.1)	*0.3694*	*0.0111*	*0.0874*
Physician	17.1 (10.9)	19.6 (10.6)	21.3 (10.2)	18.4 (10.8)	*0.0921*	*0.0226*	*0.3858*
Disease	7.1 (6.4)	7.6 (5.8)	8.1 (5.1)	7.4 (6.1)	*0.6257*	*0.3474*	*0.6084*
Sports or leisure activities	8.5 (6.3)	9.0 (6.0)	10.4 (5.5)	8.9 (6.1)	*0.5725*	*0.0585*	*0.1841*

^*^Generalized linear mixed models with spa therapy resorts as a random effect and adjusted for sex, age and number of comorbidities.

^†^Statistical test could not be used because of small numbers in stage II/III obesity group.

### Physical activity level

The IPAQ PA level and continuous score are described in Table 3. The PA level significantly differed among BMI groups (p = 0.0219). The proportion of participants with low and moderate PA level increased with BMI gradation and the proportion with high PA level decreased with BMI gradation. The BMI groups significantly differed by IPAQ continuous scores in MET-min/week: moderate activity (p = 0.0051), walking (p = 0.0201), and total activity (p = 0.0002). The walking and total activity continuous scores decreased with BMI gradation. Time spent sitting significantly differed among groups (p = 0.0025).

The IPAQ moderate activity, walking and total activity continuous scores, such as time spent sitting, significantly differed between overweight and stage II/III obesity groups and between stage I and stage II/III obesity groups.

### Fear and beliefs concerning KOA

The KOFBeQ scores are described in Table 3. The total score, daily activity score and physician score significantly differed among BMI groups (p = 0.0204, p = 0.0389 and p = 0.0413, respectively), with significantly higher scores for the stage II/III obesity group. The disease and sports or leisure activities scores did not differ between groups.

## Discussion

This study describes the clinical consequences of KOA severity in a KOA population by level of obesity. We found a graded relation between obesity stages and clinical consequences of KOA. Indeed, the results showed a progressive increase between degree of obesity based on BMI and clinical consequences. Participants with higher BMI had higher pain scores, were more disabled and reported more often anxiety and depression, which agrees with previous studies^22^. In addition, the higher the obesity stage, the less the participant performed PA, which increases the risk of leading a sedentary lifestyle.

The relationship between obesity severity and OA onset has been largely described in the literature. But to our knowledge, this is the first study to describe the clinical consequences of KOA by degree of obesity. Indeed, most studies have determined these two components in all obese people but not by BMI category. Thus, given the size of our sample, we could distinguish overweight individuals from those with grade I or II obesity and higher. Another strength of this study is the amount of data collected. Indeed, a large number of clinical variables were analyzed, which provided a broader picture when assessing daily life consequences on KOA.

The three obesity groups did not differ in location of pain. These findings agrees with the hypothesis of a “chronic micro-inflammatory state” that in conjunction with weight could play a major role in the initiation and perpetuation of OA^23^. Weight loss would be the best way to decrease chronic inflammation by reducing inflammatory mediators^24^ and also reduce mechanical load on bearing joints. Individuals with high BMI are at increased risk of metabolic syndrome, which is based on the co-occurrence of multiple risk factors (hypertension, high lipid levels) or type 2 diabetes mellitus or coronary heart disease^25,26^. Insulin resistance and micro-inflammation play a role in the development of chronic lesions. Micro-inflammation involved in the initiation and perpetuation of KOA is linked to level of insulin resistance^23^, and insulin resistance is associated with the location and proportion of fat mass^27^. Losing fat mass could play a major role in decreasing this micro-inflammation and reducing the clinical consequences of KOA.

We found the same gradual response between BMI and VAS pain score in the last 24 hours, which suggests higher pain for people with severe obesity and lower pain for overweight people. This finding may explain why weight loss may be directly associated with pain level^28^ and that the strategic approach to decrease pain could allow individuals to do more exercise. A recent study showed that in women with OA, disease-related pain was positively associated with cortisol production, particularly with greater pain intensity^29^. Pain is a potential stressor and activator of the hypothalamic-pituitary-adrenal axis, which has been related to increased visceral obesity^30^.

Strategies to control pain is a great part of the therapeutic proposition because it is a barrier to weight loss and PA. The psychological impact of obesity can be a barrier to PA and associated with severity of obesity. It could explain the association between BMI and the frequency of reported anxiety or depression. Strategies to increase the level of PA cannot be the same with different psychological profiles^31^, which emphasizes the need for personalized medicine.

PA or rehabilitation is widely recognized as one of the first non-pharmacological lines of treatment for OA and is recommended for all patients^12^. This study demonstrated that disability in patients with KOA, based on WOMAC function score, was associated with severity of obesity. With increasing obesity stage, OA increasingly led to altered function, thereby reducing the amount of PA performed, even though PA helps to improve function. Proposing PA as the first treatment aims to decrease fat mass, increase insulin sensitivity and decrease pain and micro-inflammation. However, we cannot expect severely obese individuals to do the same amount of PA as other people because they require psychological reinsurance. We found presence of anxiety and depression associated with BMI severity. This mental association with BMI severity should be more explored in future research, using validated instruments such as the Hospital Anxiety and Depression Scale (HADS)^32^ that evaluated the severity of anxiety and depression symptomatology. Obesity severity might be a stress factor recognized as a cardiovascular risk.

Concerning the representativeness of our sample, the individuals we studied are representative of samples studied in the literature^32^ and because of the high number of participants (n = 391), many different phenotypes of KOA were included. Indeed, the mean age was 67.3 years, and 71% were women. The mean WOMAC function score was 38.5. Our population was close in terms of age, sex and function to the 915 patients in the Tubach *et al*. study^32^.

The main limitation of our study is that the level of PA was based on declarative and subjective reports. This could lead to discuss validity and significance of our results. However IPAQ is an international validated questionnaire and assesses both PA and sedentary time,^33^ although it overestimates PA^34^. We found a linear relation between BMI and PA, but the relation may be overestimated^35^. Assessing the level of PA with objective measures such as an accelerometer would be interesting but still expensive. The fact that other parameters, such as OA duration, are self-reported could be a limitation of the study. Comorbidities and joint replacement could be considered reliable when reported by the patient. Knee OA criteria based on ACR criteria were verified by the physician in charge of the patient before starting the program in the spa resort for limiting this bias. Parameters such as OA duration did not significantly differ among groups. Another limitation concerns a reverse causality relation between outcomes and overweight and obesity. It is probably better to consider this as a single association rather than a real reverse causality. It is possible that our results were predictable but to our knowledge the relationship between obesity severity and PA level and several other patients reported outcomes had never been definitively demonstrated before. By the way, it is really important for every day practice to take in count obesity severity regarding PA management and rehabilitation^36^ for OA management.

Other objective data are missing in this study and would be pertinent to screen, such as body composition with distribution of mass. PA level depends on obesity severity, but the relation between fat mass index and PA could be assessed.

## Conclusion

We found level of obesity directly associated with clinical consequences of KOA, with a gradual dose–response relation by increasing BMI. High PA level was associated with low BMI and contributed to preventing the clinical consequences of KOA. The role of body fat mass in terms of clinical benefits of PA could be studied.
